# Social Mindfulness and Psychosis: Neural Response to Socially Mindful Behavior in First-Episode Psychosis and Patients at Clinical High-Risk

**DOI:** 10.3389/fnhum.2019.00047

**Published:** 2019-02-13

**Authors:** Imke L. J. Lemmers-Jansen, Anne-Kathrin J. Fett, Niels J. Van Doesum, Paul A. M. Van Lange, Dick J. Veltman, Lydia Krabbendam

**Affiliations:** ^1^Section of Educational Neuroscience, Vrije Universiteit Amsterdam, Amsterdam, Netherlands; ^2^Section Clinical, Neuro- and Developmental Psychology, Vrije Universiteit Amsterdam, Amsterdam, Netherlands; ^3^Department of Psychology, City, University of London, London, United Kingdom; ^4^Department of Experimental and Applied Psychology, Vrije Universiteit Amsterdam, Amsterdam, Netherlands; ^5^Social and Organisational Psychology, Leiden University, Leiden, Netherlands; ^6^Neuroscience Campus Amsterdam, Vrije Universiteit Amsterdam, VU Medical Center Amsterdam, Amsterdam, Netherlands

**Keywords:** social mindfulness, trust, first-episode psychosis, clinical high-risk, fMRI, mentalizing, reward

## Abstract

**Background:** Psychosis is characterized by problems in social functioning and trust, the assumed glue to positive social relations. But what helps building trust? A prime candidate could be social mindfulness: the ability and willingness to see and consider another person’s needs and wishes during social decision making. We investigated whether first-episode psychosis patients (FEP) and patients at clinical high-risk (CHR) show reduced social mindfulness, and examined the underlying neural mechanisms.

**Methods:** Twenty FEP, 17 CHR and 46 healthy controls, aged 16–31, performed the social mindfulness task (SoMi) during fMRI scanning, spontaneously and after the instruction “to keep the other’s best interest in mind.” As first of two people, participants had to choose one out of four products, of which three were identical and one was unique, differing in a single aspect (e.g., color).

**Results:** FEP tended to choose the unique item (unmindful choice) more often than controls. After instruction, all groups significantly increased the number of mindful choices compared to the spontaneous condition. FEP showed reduced activation of the caudate and medial prefrontal cortex (mPFC) during mindful, and of the anterior cingulate cortex (ACC), mPFC, and left dorsolateral prefrontal cortex (dlPFC) during unmindful decisions. CHR showed reduced activation of the ACC compared to controls.

**Discussion:** FEP showed a trend toward more unmindful choices. A similar increase of mindful choices after instruction indicated the ability for social mindfulness when prompted. Results suggested reduced sensitivity to the rewarding aspects of social mindfulness in FEP, and reduced consideration for the other player. FEP (and CHR to a lesser extent) might perceive unmindful choices as less incongruent with the automatic mindful responses than controls. Reduced socially mindful behavior in FEP may hinder the building of trust and cooperative interactions.

## Introduction

Psychotic disorder is characterized by positive psychotic symptoms (e.g., delusions and hallucinations), negative symptoms (e.g., affective flattening and lack of motivation), and cognitive impairments (American Psychiatric Association, 2013). In addition, patients display problems in social functioning (Couture et al., 2006; Fett et al., 2012), which are already present before the onset of psychosis, and have also been reported in individuals at high-risk for psychosis (Yung et al., 2003; Ballon et al., 2007; Cornblatt et al., 2007; Corcoran et al., 2011; Velthorst et al., 2016a,b). One of these social impairments is reduced trust in unknown others, a common aspect of the psychosis spectrum, which is also found in individuals at genetic and clinical high-risk for psychosis. In chronic patients reduced trust seems to persist in the face of trustworthy behavior of others, possibly due to repeated negative experiences. In contrast to first episode patients and individuals at genetic and clinical high-risk, initially reduced trust can be overcome when others are trustworthy (Gromann et al., 2013; Fett et al., 2014a, 2015, 2016; Lemmers-Jansen et al., 2018a). Additionally, patients may sometimes misplace trust: patients with a first-episode psychosis did not decrease their levels of trust when confronted with an unfair partner to the same degree as healthy controls did (Fett et al., 2016). Although trust is often assumed to be the glue to positive social interactions, little is known about what it is that helps to build trust. A prime candidate could be social mindfulness. Social mindfulness is expressed as low-cost cooperative behavior, that involves the ability and willingness to see and consider another person’s needs and wishes during social decision making (Van Lange and Van Doesum, 2015). In this paper social mindfulness is explored in first-episode psychosis patients and in patients at clinical high-risk for psychosis. We investigate whether first-episode and clinical high-risk patients show reduced spontaneous socially mindful behavior, and whether they show reduced neural activation in brain areas associated with social decision making compared to controls, similar to the trust literature in these patient groups (Gromann et al., 2013; Lemmers-Jansen et al., 2018a).

Social mindfulness (SoMi) is being thoughtful of others in the present moment, and considering their needs and wishes when making a decision (Van Doesum et al., 2013; Lemmers-Jansen et al., 2018b). Perceived socially mindful behavior will promote close relationships, facilitate cooperation, and increase trust in the other person (Declerck et al., 2013; Van Doesum et al., 2013; Van Lange and Van Doesum, 2015; Dou et al., 2018). On the contrary, displays of low socially mindful behavior may elicit reduced feelings of trust in the counterpart, who in turn will behave less trusting toward the initial actor. The ability and willingness to think about preferences of and benefits for others are two core requirements for SoMi, for trust, and for positive social interactions in general. The ability, the *skill*, reflects social cognitive processes, especially mentalizing, to recognize the needs and wishes of others, to judge the other’s trustworthiness and intentions; the willingness, the *will*, reflects social motivation, the sensitivity to the intrinsic pleasurable effects of positive social interactions, to act socially mindful or to trust (Declerck et al., 2013; Lemmers-Jansen et al., 2018b). Apart from social cognition and reward, other mechanisms may also play a role, like self-representation and self-other distinction (Fonagy and Target, 2006; van Os et al., 2010). In the SoMi task participants are presented with four items, of which three are identical and one only differed in a single aspect (e.g., three green baseball caps and one yellow baseball cap). Choosing the unique item removes the option of choice for the second player. This is the socially unmindful choice. Choosing one of the three identical items still leaves the next player a choice, making it the socially mindful choice.

Previously Lemmers-Jansen et al. (2018b) have shown that making mindful decisions engaged the fronto-parietal network and when choosing unmindfully the default mode network was recruited. Mindful and unmindful choices showed an overlap of activated regions, especially in medial prefrontal cortex (mPFC) and the temporo-parietal junction (TPJ). Exclusion analysis revealed condition specific activation for mindful choices in parietal regions. Unmindful choices activated frontal regions (anterior cingulate cortex (ACC) and mPFC). The caudate was associated with mindful choices in prosocially oriented subjects, indicating a rewarding aspect of prosocial behavior. These regions are consistent with the reward, cognitive control, and social cognition systems, each of which is implicated in prosocial decision making (Declerck et al., 2013).

Patients with psychotic disorder show aberrant activation of these brain areas, which are often associated with mentalizing and reward processing (Juckel et al., 2006; Murray et al., 2008; Schilbach et al., 2016; Bartholomeusz et al., 2018). Both mechanisms have been linked to trust (Brüne, 2005; King-Casas et al., 2005; Baas et al., 2008; Marwick and Hall, 2008; Benedetti et al., 2009; Gromann et al., 2013; Billeke et al., 2015; Horat et al., 2017; Lemmers-Jansen et al., 2017). In patients at clinical high-risk for psychosis (CHR) and in unaffected siblings of patients similar social cognitive impairments are found, albeit to a lesser degree, suggesting milder impairments in high-risk populations, and a major decline with the first episode (Pinkham et al., 2007; Bora and Pantelis, 2013; Lavoie et al., 2013; McCleery et al., 2014). CHR are already in care for other psychopathology, reporting psychotic-like symptoms, but have not yet experienced (or never will) full-blown psychosis (Velthorst et al., 2009; Woods et al., 2009; van Os and Linscott, 2012; Wigman et al., 2012; van Os and Reininghaus, 2016). With the conversion to psychosis, impairments in social function increase, therefore it is important to understand the changes that occur during this transition. Investigating social interactions in patients with psychotic symptoms, first-episode psychosis patients (FEP) and CHR, who are unbiased with regard to long lasting stigma and institutionalized living can help identifying processes that decline at first onset. This may provide specific targets for intervention, to prevent or delay social decline, which is crucial for outcome prognosis and early intervention.

Isolated social cognitive skills have been successfully assessed with off-line tasks; however, they do not capture the wide range of mechanisms involved in social interactions. Real life social interactions are difficult to measure in a controlled environment, but neuro-economics provide paradigms, investigating sharing or trusting behavior in real interactions. They can capture social cognitive skills, as well as the neural processes underlying social behavior. When investigating impairments in social behavior in psychopathology, especially schizophrenia/psychosis, studying these paradigms with fMRI can advance the understanding of the neurobiology of social dysfunction (Kishida et al., 2010; Hasler, 2012; Cáceda et al., 2014; Riccardi et al., 2015). Studies have shown aberrant behavioral outcomes and neural mechanisms during trust processing in patients with psychosis (Fett et al., 2012, 2014a, 2015, 2016; Gromann et al., 2013; Lemmers-Jansen et al., 2018a). The SoMi paradigm resembles everyday interpersonal situations by involving very little costs (c.f. giving compliments or making nice gestures), and low-level cooperation, as reflected in a straightforward choice for an item, whereas trust can be seen as high-level cooperation, with more at stake, including risk, and building a model about the counterpart. Furthermore, unlike other neuro-economical paradigms, where the pay-offs for the player and the other person are usually very clear, in the SoMi task participants have to recognize or see what others want, and how their actions influence the outcomes for others. Thus, the situation has to be recognized as a social one, with all the associated demands and opportunities. This realization is an intricate part of the construct.

The current study sets out to investigate behavioral and neural mechanisms of spontaneous socially mindful decisions in FEP and CHR patients. Given that patients show impairments in reward processing and social cognitive skills, including taking the perspective of the other person, we hypothesized that (1) FEP will opt more often for individual gain (the unique item), and therefore spontaneously make more unmindful choices compared to controls. Given the straightforward nature of the task, we further hypothesized that (2) FEP, similar to controls, make more socially mindful choices after being asked to keep the other’s best interest in mind. Given the evidence for altered brain activation during social decisions and impairments in reward processing and mentalizing in patients, we hypothesized that (3) FEP will show reduced activation of the caudate during spontaneous mindful choices, and generally less activation in mPFC and TPJ compared to controls. With regard to CHR, we hypothesized that they will show (4) an intermediate behavioral performance compared to FEP and controls (Giuliano et al., 2012; Thompson et al., 2012; Lemmers-Jansen et al., 2018a), and intermediate neural activation compared to FEP and controls. Additionally, associations of positive and negative symptoms, and paranoia with behavioral and neural outcomes are explored, based on the association between paranoia and reduced trust, and mixed outcomes in the trust game literature (Gromann et al., 2013; Fett et al., 2014b; Lemmers-Jansen et al., 2018a).

## Materials and Methods

### Subjects

Twenty-nine young adolescents with a first psychotic episode (FEP), aged 16–22 were recruited in the Amsterdam area. Additionally, 18 patients at clinical high-risk for developing psychosis (CHR) and 52 controls, aged 16–31 were recruited in the Amsterdam and The Hague area. All patients were contacted through their treating clinicians at the academic medical center Amsterdam (AMC), the Amsterdam early intervention team psychosis (“Vroege Interventie Psychose” or VIP team), and PsyQ The Hague. FEP were diagnosed at the AMC, according to the DSM-IV criteria (American Psychiatric Association, 2000), and included within 18 months of the diagnosis (*M* = 5.6 months). Thirty percent was unmedicated, 55% was on atypical antipsychotic medication, and 15% on other psychotropic medication. FEP illness ranged from hospitalized to reentering work and society living, with symptoms ranging from mildly to markedly ill, and one severely ill patient (Leucht et al., 2005). CHR were help seeking individuals that were referred to PsyQ by their general practitioners or other mental health institutions. After an initial diagnosis based on their complaints, all new admissions (between age 14–35) were screened for an “at-risk mental state” (ARMS) with the Comprehensive Assessment of At-Risk Mental States [CAARMS; (Yung et al., 2005)], a semi-structured interview that assesses psychotic experiences in the last year before assessment. Additionally, patients had to display marked problems in socially useful activities (work and study), relationships, and self-care, indicated by a score below 55 on the Social and Occupational Functioning Assessment Scale [SOFAS; mean score 46.9; (Goldman et al., 1992; Morosini et al., 2000)], see also (Rietdijk et al., 2012). CHR were included within 1 year after CAARMS assessment (*M* = 4.8 months). Symptoms of depression and anxiety are often the primary presenting complaints of CHR patients, rather than (subclinical) psychotic symptoms (Modinos et al., 2014). Similar to other CHR samples (Woods et al., 2009; Kelleher et al., 2012; Morrison et al., 2012; Wigman et al., 2012; Fusar-Poli et al., 2014), the current CHR sample had comorbid diagnoses of anxiety (5), personality (3), eating (2) and mood (2) disorders, trauma (2), and ADHD (3). Exclusion criteria for both patient groups were primary diagnosis of mood disorders, comorbidity with autism spectrum disorder (ASD) and an IQ < 80, information provided by their primary clinicians, based on the initial assessment and diagnosis. And for the healthy control group this was a family history of psychiatric disorders, ASD and an IQ < 80, as was assessed with a questionnaire and by recruiting participants from regular educational institutes. All participants were fluent in Dutch. We excluded nine FEP, one CHR, and six controls from analyses due to invalid or missing data. The remaining sample consisted of 20 FEP, 17 CHR, and 46 controls. The first study on the neural mechanisms of social mindfulness was based on the same sample of healthy controls (Lemmers-Jansen et al., 2018b). This research was approved by the Ethics Committee of the VU Medical Center Amsterdam.

### Measures

#### Social Mindfulness Paradigm (SoMi Task)

**FIGURE 1:**
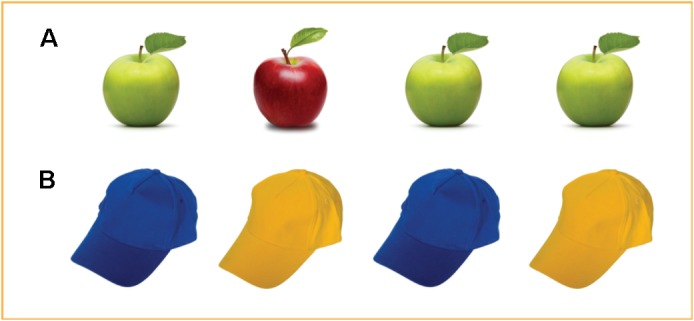
Example trials of the Social Mindfulness task (SoMi), displaying **(A)** an experimental trial (3:1 ratio presentation) where the participant’s choice can influence the choice options of the other player; and **(B)** a control trial (2:2 ratio presentation) where the choice has no social consequences. The stimulus was displayed for 5000 ms, followed by an inter-stimulus interval (0, 1000, or 2000 ms). Reproduced from (Lemmers-Jansen et al., 2018b).

The SoMi task consisted of a dyadic game in which the participant and a fictitious other (someone “who you don’t know and are not likely to meet in the near future”) repeatedly choose what to take from a set of four similar products [identical task characteristics as in Lemmers-Jansen et al. (2018b)]. One of these products was unique in a single aspect, whereas the other three were identical, for example one red among three green apples (1:3 ratio; see Figure 1). Participants were instructed that they would always choose first, and that chosen items would not be replaced. Choosing an identical item would leave the next person a choice, and was scored as socially mindful; taking away the unique item would limit this other person’s choice, and was scored as socially unmindful. Each of the experimental trials featured different products. All products were low in value, e.g., pens, water bottles, etc. We added control trials as a baseline measure to the analyses, which displayed the items in a 2:2 ratio in which the participant’s choices would have no social consequences (see Figure 1).

The SoMi paradigm was administered twice. In the first round (spontaneous condition), participants only received the general information that someone else would choose after them. In the second round (instructed condition), participants received the additional instruction to “keep the best interest of the other person in mind” (cf. Van Doesum et al., 2013, Studies 1a–c). This round was added to check if lower scores on social mindfulness are the result of a lack of ability to understand how one’s own behavior affects the other player. Each round consisted of 24 experimental trials, with one unique versus three identical items (e.g., one red and three green apples); 24 control trials, offering two pairs of identical items (e.g., two blue and two yellow baseball hats), and 12 low-level baseline trials, where participants passively watched a blank screen. Each trial had a duration of 5000 ms. A final score of social mindfulness was computed. This SoMi-index is the proportion of socially mindful answers, varying from 0 (only socially unmindful choices) to 1 (only socially mindful choices).

#### Positive and Negative Syndromes Scale (PANSS)

The well validated 30-item PANSS semi-structured interview was used for rating symptoms in the 2 weeks prior to testing. The PANSS distinguishes between positive, negative, and general symptoms (Kay et al., 1987). The item P6 was used as an indication for paranoia. Items are scaled on a 7-point Likert scale, ratings 3 and higher indicating clinical values. All FEP and 13 CHR completed the interview.

#### Wechsler Adult Intelligence Scale (WAIS) Vocabulary

A subtest of the WAIS-III (Wechsler, 1997) was included as a proxy for intelligence. The vocabulary subscale, a measure of verbal comprehension, consisted of 33 words that had to be defined or described by the participants (e.g., winter, catastrophe, and reckless). Answers were either fully correct (2 points), partially correct (1) or wrong (0). After six consecutive 0 scores, the test was discontinued.

### Procedure

All participants provided general informed consent; patients also signed a form that allowed the researchers to obtain additional patient data from their care giving institution. After signing the consent forms, participants completed several pen and paper questionnaires, followed by two computer-administered tasks. Both patient groups were assessed with the PANSS. Medication use was assessed with the pre-scanning questionnaire, a questionnaire pertaining to the safety procedure for scanning. Subsequently participants were scanned for about an hour. For patients, extra time was needed to guide them into the scanner, comfort them and to ensure they understood the tasks. Therefore, we planned 15 min extra for them. First, all participants performed an unrelated task [the Trust Game, see (Lemmers-Jansen et al., 2018a)]. Next the structural scan was made, during which participants could relax, while watching a movie if they wanted. The SoMi task was the second task participants performed in the scanner. Two rounds of the SoMi task were played as described above, each round lasting 6 min. Instructions for the task were given in the scanner, immediately prior to the task. Four practice trials were completed before the task started to ensure that instructions were clear. Instructions for the second round were given visually and orally, while scanning was paused. Scanning sessions ended with a resting state scan. After scanning participants received an image of their structural brain scan, 25€ for participation and travel costs were reimbursed.

### fMRI Data Acquisition

fMRI data were obtained at the Spinoza center Amsterdam, using a 3.0 T Philips Achieva whole body scanner (Philips Healthcare, Best, Netherlands) equipped with a 32 channel head coil. A T2^∗^ EPI sequence (TR = 2, TE = 27.63, FA = 76.1°, FOV 240 mm, voxel size 3 × 3 × 3, 37 slices, 0.3 mm gap) was used, resulting in 185 images per condition. A T1-weighed anatomical scan was acquired for anatomical reference (TR = 8.2, TE = 3.8, FA = 8°, FOV 240 mm^∗^188 mm, voxel size 1 × 1 × 1, 220 slices).

### Data Analysis

#### Behavioral Data

Demographic and behavioral data were analyzed using Stata 13 (StataCorp, 2013) with regression analyses and chi-square tests. For behavioral outcomes, *t*-tests and regression analyses were used. Analyses included spontaneous choices and choices after instruction, and were controlled for age and gender as *a priori* confounders, and for WAIS Vocabulary, to avoid potential confounding effects of group differences. To examine whether the results were influenced by general cognitive impairment in patients, all analyses were repeated without WAIS Vocabulary.

#### Imaging Data

Imaging data were analyzed using Statistical Parametric Mapping (SPM8; Wellcome Trust Centre for Neuroimaging, London, United Kingdom). Functional images for each participant were preprocessed with the following steps: realign and unwarp, coregistration with individual structural images, segmented for normalization to an MNI template and smoothing with a 6 mm full width at half maximum (FWHM) Gaussian kernel. At fist-level, a general linear model (GLM) was used to construct individual time courses for the onset of the presentation of the trial, and individual reaction times for the spontaneous and instructed conditions. Decision making was defined as the interval between stimulus onset and button press. In the SoMi trials (3:1 ratio) a distinction was made between the socially mindful (choosing one of the three identical items) and unmindful responses (choosing the unique item). The choices made in the spontaneous and instructed rounds were contrasted with the corresponding control trials (2:2 ratio).

At second level, a three-group factorial design was used for the main effects and group comparisons. Participants were only included in the analysis of the SoMi trials if they had at least 1/3 of the 24 responses within a response category: Participants with 1–7 unmindful responses were included only in the mindful condition, with 8–16 unmindful choices were included in both mindful and unmindful conditions, and with 17–24 only in the unmindful condition. Due to this procedure, sample size varied per condition. Mindful and unmindful responses in the spontaneous condition and mindful responses after instruction were included in the neural analyses. The unmindful condition after instruction included too few participants for reliable analyses. Analyses were controlled for age, gender, and WAIS.

Whole brain main effects of social choice (all SoMi trials, including mindful and unmindful choices; FWE corrected) over groups were calculated, to define the coordinates for the regions of interest (ROI). Regions involved in social decision making, conflict processing, and self- and other-representation, were predefined on the basis of previous neuroimaging studies (Zhu et al., 2007; Rilling and Sanfey, 2011). When activated in the whole brain analysis, peak coordinates of the predefined regions were extracted and a 10 mm sphere was built around this peak. For the bilateral caudate a 5 mm sphere was used. Whole brain results did not show activation clusters for the ACC and right insula. Coordinates for the ACC were therefore manually defined from a larger prefrontal cluster, covering the ACC; right insula coordinates were mirrored from the contralateral region. This resulted in the following ROIs: mPFC (MNI coordinates: 0, 50, 34), precuneus (9, -52, 31), ACC (3, 47, 13), and bilateral insula (33, 20, -14 and -27, 20, -14), caudate (12, 8, 13 and -12, 5, 13), TPJ (51, -52, 46 and -51, -55, 43), and dlPFC (42, 14, 49 and -39, 20, 46). A priori ROI analyses compared group activation per condition. *P*-values were Bonferroni corrected for multiple comparisons and adjusted for internal correlations, by using the Simple Interactive Statistical Analysis Bonferroni tool^1^, resulting in adjusted significance thresholds (Woudstra et al., 2013; Li et al., 2014; Lemmers-Jansen et al., 2018a). Additional whole-brain group comparisons were performed, to investigate activation outside the predefined ROIs.

## Results

### Participant Characteristics

Participant characteristics are shown in Table 1. FEP, CHR and controls did not differ significantly from each other with respect to gender, handedness, and other measures (see Table 1). However, CHR were significantly older than FEP (β = 0.56, *p* < 0.001), and controls (β = 0.40, *p* < 0.001). Furthermore, FEP scored significantly lower than CHR (β = -0.30, *p* = 0.02), and controls (β = -0.34, *p* = 0.003) on the WAIS Vocabulary scale. Between the patient groups, no significant differences were found in number of medicated participants, nor in symptom severity.

**Table 1:** Participant characteristics.

	FEP *N* = 20	CHR *N* = 17	Controls *N* = 46	Statistics
Gender (*n* male, %)	13 (65%)	7 (41%)	24 (52%)	χ^2^ = 2.12
Age (Mean/*SD*)	19.96 (1.56)	**23.78 (2.49)**	21.10 (2.72)	*F* = 11.85^∗^
WAIS (Mean/*SD*)	**32.8 (11.02)**	41.71 (12.16)	42.11 (11.26)	*F* = 4.96^∗^
Right handed *n* (%)	16 (80%)	17 (100%)	38 (83%)	χ^2^ = 4.09
Medicated *n* (%)	14 (70%)	8 (47%)		χ^2^ = 0.16
• Atypical antipsychotics (*n)*	11	–		
• Other psychotropics (*n)*	3	8		
PANSS – total (*SD*)	60.70 (15.32)	58.92 (11.84)		*F* = 0.13
• Mean severity (*SD*)	2.02 (.51)	1.96 (0.39)		
Positive – total (*SD*)	13.60 (6.0)	13.38 (2.69)		*F* = 0.02
• Mean (*SD*)	1.94 (0.86)	1.91 (0.38)		
Negative – total (*SD*)	16.80 (6.13)	13.69 (3.88)		*F* = 2.64
• Mean (*SD*)	2.40 (0.88)	1.96 (0.55)		
General – total (*SD*)	30.30 (7.73)	31.85 (6.31)		*F* = 0.36
• Mean	1.89 (0.48)	1.99 (0.39)		
P6 paranoia item (*SD*)	1.9 (1.6)	1.2 (0.4)		*F* = 2.64


*^∗^Significant group differences at p < 0.05, with the group in bold differing from the two other groups.*

*FEP, first-episode psychosis; CHR, clinical high-risk. WAIS, Wechsler Adult Intelligence Scale; PANSS, Positive and Negative Syndrome Scale.*

### Behavioral Results

#### Spontaneous Choices

Partly confirming our first hypothesis, FEP showed a trend toward spontaneously choosing the unique item more often than controls, (β = -0.22, *f*^2^ = 0.15, *p* = 0.08; see Table 2), but not than CHR (β = 0.08, *p* = 0.59). CHR did not differ significantly from controls (β = -0.12, *p* = 0.33). The difference between spontaneous mindful and unmindful choices was significant in all groups (controls: *t* = -4.0, *p* < 0.001; CHR: *t* = -2.0, *p* = 0.05; FEP: *t* = 2.1, *p* = 0.04). Note that spontaneously FEP made more socially unmindful than socially mindful choices, whereas CHR and controls made more socially mindful choices, resulting in a SoMi index under 0.5 for FEP (i.e., 0.45), and above 0.5 for CHR and controls (0.54 and 0.56, respectively).

**Table 2:** Number of choices and participants for fMRI analysis per condition by group.

Condition	FEP (*N* = 20)	CHR (*N* = 17)	Controls (*N* = 46)
*Spontaneous*			
Mindful, mean (*SD*)	10.80 (3.41)^∗^	13.06 (3.17)	13.43 (3.65)
Unmindful, mean (*SD*)	13.05 (3.40)^∗^	10.82 (3.23)	10.39 (3.64)
*Instructed*			
Mindful, mean (*SD*)	17.05 (7.19)^∗^	20.12 (4.05)	20.76 (4.41)
Unmindful, mean (*SD*)	6.95 (7.19)^∗^	3.76 (4.01)	3.24 (4.41)
SoMi-index	0.45 (0.14)^∗^	0.54 (0.13)	0.56 (0.15)
**Number of participants for fMRI analysis**			
Social decision	20	17	46
Spontaneous mindful	18	16	43
Spontaneous unmindful	20	15	37
Mindful after instruction	18	17	45
Unmindful after instruction	8	3	8


*^∗^p = 0.08, FEP differing at trend level from controls. FEP, first-episode psychosis; CHR, clinical high-risk; SoMi index, proportion of socially mindful choices*.

#### Choices After Instruction

After instruction FEP showed the same trend to choose the unique item more often than controls (β = -0.22, *p* = 0.08), but not than CHR (β = 0.12, *p* = 0.45), and CHR did not differ significantly from controls (β = -0.08, *p* = 0.51). After instruction all groups made significantly more socially mindful than socially unmindful choices (all *t*’s < -4, all *p*’s < 0.001). Additionally, all groups significantly increased the number of mindful choices compared to the spontaneous condition (all *t*’s < -3.5, all *p*’s ≤ 0.001), indicating that the manipulation was effective. The difference at trend level between FEP and controls in the number of socially mindful choices persisted after instruction, showing no significant group differences in the number of socially mindful choices after instruction similarly (β = -0.05, *p* = 0.67). The CHR group performed in between FEP and controls, resembling the control group most.

Additional analyses without WAIS as a covariate showed the same results, with similar significance levels, and comparable medium to large effect sizes. However, the trend result of FEP choosing more often the unique option than controls now reached significance, in both spontaneous choices (β = -0.27, *p* = 0.02) and choices after instruction (β = -0.24, *p* = 0.02).

### Symptoms

Associations between the paranoia item, positive and negative symptoms and behavioral outcomes were investigated in FEP and CHR. Group-by-symptom interactions on spontaneous and instructed choices were non-significant (all |β’s| < 1.4, *p*’s > 0.21), as were the group-by-symptom interactions on increase of mindful choices after instruction (β’s < 0.67, *p*’s > 0.59). Removing the interactions from the model showed an inverse main effect at trend level of negative symptoms on increase of mindful choices after instruction (β = -0.33, *p* = 0.08), indicating that patients with higher levels of negative symptoms showed a smaller increase of mindful choices after instruction than patients with less negative symptoms.

### fMRI Results

#### ROI Analyses

Analogous to our previous study (Lemmers-Jansen et al., 2018b), participants were only included in a condition when they had at least 1/3 of the decisions within that particular condition (see section “Imaging Data”). Due to this procedure, sample size varied per condition, see Table 2.

To determine the coordinates for the predefined ROI, whole brain analysis of social choice over all trials and all groups were conducted (see Table 3). Regions and coordinates used for ROI analyses are marked in bold font. ROI analyses were performed with 11 predefined ROIs. ROI analysis outcomes are presented in Table 4. During spontaneous mindful choices, the caudate was less activated in FEP than controls; and the mPFC was less activated in FEP than both CHR and controls. During spontaneous unmindful choices controls activated the ACC significantly more than both CHR and FEP, and controls showed more activation in the mPFC and the left dlPFC than FEP. Summarizing, most activation was found in controls, with CHR performing in between FEP and controls. Mindful choices after instruction yielded no significant group differences. Replication of the analyses without WAIS Vocabulary as covariate yielded similar significance levels in the same ROIs as displayed in Table 4.

**Table 3:** Whole brain main effects of social choices, including all SoMi trials, regardless of choice, over all groups.

Region	Hemisphere	MNI coordinates			Cluster size *k*	*z*
		*X*	*Y*	*Z*		
mPFC	L	–6	38	46	807	7.32
**mPFC**	**R**	**0**	**50**	**34**		7.28
mPFC	R	12	44	46		6.84
mPFC	R	6	68	7	6	5.42
mPFC	R	9	62	28	2	5.14
Inferior frontal gyrus	L	–51	17	7	21	5.65
**dlPFC**	**R**	**42**	**14**	**49**	89	6.94
**dlPFC**	**L**	–**39**	**20**	**46**	66	6.02
Inferior orbitofrontal gyrus	R	36	23	–11	144	6.88
Inferior orbitofrontal gyrus	R	48	35	–11		6.57
Middle orbitofrontal gyrus	R	39	56	–2		5.37
Middle orbitofrontal gyrus	L	–42	50	–2	1	4.74
**Insula**	**L**	–**27**	**20**	–**14**	30	6.16
Inferior orbitofrontal gyrus	L	–33	20	–23		5.40
Inferior orbitofrontal gyrus	L	–48	38	–8	3	5.39
Inferior frontal operculum	R	57	20	13	58	7.20
Superior frontal gyrus	L	–21	59	22	2	4.96
Middle temporal gyrus	R	63	–43	–5	29	5.94
Middle temporal gyrus	L	–54	–22	–11	42	5.45
Middle temporal gyrus	L	–63	–28	–5		5.40
Middle temporal gyrus	L	–48	–31	–5		5.32
Middle temporal gyrus	R	63	–13	–14	1	4.74
Superior temporal pole	L	–45	20	–14	14	5.78
Inferior temporal gyrus	L	–48	–1	–32	3	4.94
Angular gyrus	R	57	–61	34	416	>7.7
**TPJ**	**R**	**51**	–**52**	**46**		6.94
**TPJ**	**L**	–**51**	–**55**	**43**	342	7.68
Angular gyrus	L	–54	–64	25		6.91
Angular gyrus	L	–42	–67	46		6.69
**Caudate**	**R**	**12**	**8**	**13**	13	5.55
**Caudate**	**L**	–**12**	**5**	**13**	4	5.00
Mid cingulum	L	–3	–22	34	312	6.58
**Precuneus**	**R**	**9**	–**52**	**31**		6.07
Precuneus	R	3	–67	34		5.64


*Regions displayed in bold font correspond with predefined regions of interest (ROI). MNI, Montreal Neurological Institute; mPFC, medial prefrontal cortex; dlPFC, dorsolateral prefrontal cortex; TPJ, temporo-parietal junction; L, left; R, right. Regions and coordinates for the ROI are displayed in bold font. Two additional ROI were defined: Anterior cingulate cortex (ACC): 3, 47, 13, based on the large prefrontal cluster, and right insula: 33, 20, –14, based on mirroring the left insula. Analyses were FWE corrected, at p < 0.05*.

**Table 4:** Region of interest analysis outcome per condition in the SoMi paradigm.

	ROI	CHR > FEP		Con > FEP		Con > CHR	
		*t*	*p*	*t*	*p*	*t*	*p*
Spontaneous mindful^∗^	mPFC	1.74	0.043ˆ	2.30	0.012		
	Right caudate			1.84	0.035		
Spontaneous unmindful^∗∗^	ACC			1.93	0.029	1.85	0.34
	Left dlPFC			2.01	0.024		
	mPFC			1.86	0.034		


*^∗^Significance level of p = 0.039, Bonferroni corrected, adjusted for internal correlation. ^∗∗^Significance level of p = 0.042, Bonferroni corrected, adjusted for internal correlation. ˆBordering significance. ROI, region of interest; CHR, clinical high-risk; FEP, first-episode psychosis; Con, healthy controls; mPFC, medial prefrontal cortex; ACC, anterior cingulate cortex; dlPFC, dorsolateral prefrontal cortex*.

#### Exploratory Whole Brain Analyses

Additional whole brain analyses on group differences per SoMi condition revealed no group differences surviving the FWE cluster correction. To verify that all three groups showed similar brain activation, a global-null analysis was performed. Results are shown in Supplementary Table S1, and indicate similar networks as described in our previous paper with a partly overlapping sample (Lemmers-Jansen et al., 2018b). During spontaneous unmindful choices, however, this analysis also revealed additional activation in the ventrolateral prefrontal cortex, caudate, and insula.

#### Associations With Symptoms

Analyses showed no significant associations between contrast estimates and symptoms. Contrast estimates of the significant ROI were associated with positive and negative symptoms. In the mPFC during mindful choices (the only ROI with significant differences between the two patient groups), no significant group-by-symptom interactions were found (positive: β = -0.69, *p* = 0.6; negative: β = 1.05, *p* = 0.34; paranoia: β = -0.13, *p* = 0.95). After removing the interaction from the model, symptoms did not show a significant main effect on mPFC activation. In the ROIs where patient groups differed significantly from to controls, i.e., the right caudate during mindful choices, and the ACC, mPFC and left dlPFC during unmindful choices, the only significant association was in the dlPFC with paranoia, indicating increased activation with increasing paranoia (β = 0.49, *p* = 0.029).

## Discussion

The purpose of the present research was to examine the behavioral outcomes and neural substrates of socially mindful and unmindful choices, in a clinical high-risk (CHR) and first-episode psychosis (FEP) sample. The results showed a trend toward more spontaneously unmindful choices in FEP compared to the CHR and control group, but a similar increase of socially mindful choices after instruction across the three groups, indicating the ability for socially mindful behavior when prompted. At the neural level FEP showed decreased activation in the caudate compared to controls when making socially mindful choices, possibly suggesting reduced sensitivity to the rewarding aspects of social mindfulness. Additionally, reduced activation in the mPFC, ACC and dlPFC was found in FEP during unmindful choices, suggesting that FEP might perceive unmindful choices as less incongruent with the automatic mindful responses than controls. Scores for CHR were in between FEP and controls.

### Behavioral Results

In partial support of our hypothesis, we found a marginal effect showing that FEP tended to make spontaneously more socially unmindful choices than controls. This result became significant when analyses were run without the covariate WAIS Vocabulary, a proxy for intelligence. Despite the visual nature of the task, social mindfulness seems to depend on cognitive ability. The small reduction of effect size, however, suggests only a minimal confounding effect. FEP opted more often for the unique than for the non-unique option, with a mean proportion of social mindfulness of 0.45, while the other groups chose more often the non-unique item (mean proportion CHR: 0.54; controls: 0.56). Other studies have shown that the mean proportion of social mindfulness toward strangers converges around 0.67 (Van Doesum et al., 2013; Van Lange and Van Doesum, 2015). Social mindfulness tends to be greater in prosocially orientated individuals; when the other player has a trustworthy face, is an in-group member, or is someone liked (Van Doesum et al., 2013, 2016) and when the second person is perceived as lower in social class than the participant (Van Doesum et al., 2017). When interacting with a friend, social mindfulness also increases (Van Lange and Van Doesum, 2015; Van Doesum et al., 2016). However, with a foe or an outgroup member, the proportion of socially mindful choices decreases to around 0.45, which could be labeled as social hostility (Van Doesum et al., 2016). FEP showed a similarly low proportion of social mindfulness, suggesting that they were spontaneously less inclined to consider the interest of the partner. This finding is of theoretical interest because it indicates that psychotic disorder is also linked to differences in spontaneous low-cost cooperation. As noted earlier, social mindfulness is causally linked to maintaining or enhancing trust: Greater social mindfulness yields greater trust in the recipient of socially mindful behavior. And especially, more social unmindfulness undermines trust (see Van Doesum et al., 2013; Dou et al., 2018), in that the negative consequences (ending up having no choice) tend to outweigh positive consequences in terms of attention, and of what people recall and reciprocate (Van Lange et al., 2002). Whether SoMi is sensitive to interventions remains to be determined in future research. We suggest that the SoMi task has some features, such as the emphasis on perspective taking and giving small favors to others, that might make it suitable for intervention purposes. However, there is a big differences between instructing social mindfulness and actually expressing it in a spontaneous manner in real life situations.

Contrary to our hypothesis, the mean SoMi score of CHR was not between FEP and controls, but CHR displayed a similar level of spontaneous socially mindful behavior as controls. Low level cooperation therefore seems to be still intact in CHR, contrary to the higher level trust processing, where CHR showed reduced levels of baseline trust, similar to FEP [cf. (Lemmers-Jansen et al., 2018a)]. Confirming our hypothesis, though, all groups showed a similar increase of socially mindful choices when instructed to keep the other’s best interest in mind, indicating that low levels of social mindfulness in FEP did not reflect an inability to understand the impact of their behavior on the partner, but rather a reduced tendency to consider other’s perspective spontaneously. These findings suggest an impact of the first psychotic episode on spontaneous socially mindful behavior. This tentatively suggests that reduced socially mindful behavior in FEP may affect social interactions with other people, which may fail to evolve according to the positive reciprocity that characterizes ‘typical’ patterns of interactions, if not made explicitly clear. However, similar to observations of initially reduced trust in FEP, our findings show that this pattern can be overcome through positive feedback (Lemmers-Jansen et al., 2018a).

### Neural Results

The analyses of the brain activation corroborated the behavioral findings that FEP were able to act socially mindfully when prompted: No group differences in brain activation were found in the mindful condition after instruction.

As hypothesized, FEP showed reduced activation of the caudate compared to controls. Reduced caudate activity during socially mindful choices might reflect reduced feelings of reward when leaving the other the option, setting aside one’s own preferences. Impairments in reward processing in psychosis have frequently been reported (Juckel et al., 2006; Waltz et al., 2010; Strauss et al., 2013). Neuro-economic research using the trust game in chronic patients similarly showed reduced caudate activity during positive social interactions (Gromann et al., 2013; Brown et al., 2014). The current findings suggest that reduced reward processing may extend to socially mindful behavior. When social interactions or doing good are not perceived as inherently rewarding (Higgins and Scholer, 2009), FEP will less likely engage in other regarding interactions. Furthermore, in line with our hypothesis, activation of the mPFC, one of the regions previously shown to be engaged in both mindful and unmindful choices (Lemmers-Jansen et al., 2018b) was reduced in FEP compared to controls (and CHR) in both choice types. The mPFC is involved in many aspects of social and general cognition, such as mentalizing, learning, memory, cognitive control, decision making, predicting valence and timing of expected outcomes of an action, reward anticipation and salience, and in processing emotions (Ridderinkhof et al., 2004; Frith and Frith, 2006; Van Overwalle and Baetens, 2009; Ziauddeen and Murray, 2010; Forster and Brown, 2011; Euston et al., 2012; Cáceda et al., 2014). Considering this range of functions in the context of the current paradigm, reduced mPFC activation might indicate that FEP consider the consequences of their decisions for the other player less than controls and CHR. It is important to consider that reduced mPFC activation in both decision types might also reflect general and not task related reduced activity of this region, inherent to psychosis patients (Sugranyes et al., 2011). Contrary to our predictions FEP did not display reduced TPJ activation in socially mindful, nor in socially unmindful choices. As hypothesized, reward and mentalizing mechanisms may play a role in social mindfulness. This is supported by the activation of mPFC and caudate during mindful decisions. No differences were found between groups in the ROIs that are typically related to self-perception and self-other representation (insula, precuneus, and TPJ), suggesting that these mechanisms are unlikely to play a role. However, the association between these mechanisms and social mindfulness warrants further investigation with additional measures.

When making socially unmindful decisions, FEP showed reduced activation of mPFC, ACC, and dlPFC, the latter being associated with the paranoia score. Reduced mPFC activation in both spontaneous choice options could indicate reduced anticipation of thoughts and feelings of others (Frith and Frith, 2006), although other process might also play a role in socially unmindful decisions. Alternatively, after instruction to mind the other’s best interest, no differences in neural activation were present, suggesting that FEP only show impairments in spontaneously anticipating the feelings of others, but follow instructions similar to controls. The ACC and dlPFC are, among many cognitive processes, involved in cognitive control and conflict processing (MacDonald et al., 2000; Milham et al., 2003; Badre and Wagner, 2004; Ridderinkhof et al., 2004; Mitchell et al., 2009). Based on predominantly prefrontal activation during socially unmindful decisions, when contrasted with socially mindful decisions, we previously concluded that in healthy subjects socially unmindful decisions seemed to be more deliberate, requiring cognitive control, whereas socially mindful decisions were the more automatic response (Lemmers-Jansen et al., 2018b). Reduced ACC and dlPFC activation in FEP might therefore indicate that FEP perceive socially unmindful choices as less incongruent or deliberate, and less effortful. The association of dlPFC activation and paranoia warrants further investigation.

In contrast to FEP, CHR showed no impairments in reward processing areas, possibly explaining the intact spontaneous socially mindful behavior. No differences in mentalizing areas were found, suggesting normal functioning of this mechanism. CHR showed less reduction in activation than FEP, especially in prefrontal areas [see also (Morey et al., 2005; Broome et al., 2010; Schmidt et al., 2013)]. These results only partly confirm our hypothesis of intermediate neural activation compared to FEP and controls. Reduced ACC activation compared to controls during unmindful choices might indicate, similar to FEP, that CHR also perceive unmindful choices as less incongruent or effortful than controls. Differential neural activation in patients at-risk despite similar behavioral performance was previously found, although activation in CHR was often increased (Morey et al., 2005; Marjoram et al., 2006; Seiferth et al., 2008; Brüne et al., 2011; Derntl et al., 2015).

The frequency of spontaneous socially mindful behavior appeared to be independent of symptom severity, but reduced after a first psychotic episode. Future research could investigate this behavior in chronic illness, testing whether spontaneous socially mindful behavior further declines with illness duration. Interestingly, more negative symptoms were associated with less increase of mindful choices in both patient groups after instruction. Negative symptoms have been related to avolition, reduced social motivation, and poor social functioning and cognition in both FEP and CHR (Milev et al., 2005; Voges and Addington, 2005; Chan and Chen, 2011; Corcoran et al., 2011; Meyer et al., 2014). However, they are not related to reduced spontaneous socially mindful behavior, but to reduced changes in socially mindful behavior after being told to mind the other’s best interest, possibly indicating reduced propensity to set aside their own preferences for the benefit of others.

### Limitations and Future Directions

Several limitations should be considered. First, the size of the sample was modest, especially of the CHR group. Results should therefore be considered as a first step investigating socially mindful decision making in these patients, demanding replication and extension in future research. Larger samples would permit subtyping of FEP and comparing CHR that transitioned to psychosis with non-converters, yielding more information about social mindfulness and its underlying mechanisms in patient populations. Furthermore, only one CHR patient transitioned to psychosis, 1 year after participating in this study. This could raise questions about the representativeness of the sample. However, our sample was comparable to other samples in terms of comorbidities (Woods et al., 2009; Corcoran et al., 2011; Morrison et al., 2012; Fusar-Poli et al., 2014; Modinos et al., 2014; Ising et al., 2016), and participants were assessed with the CAARMS, and included when scoring below 55 on the SOFAS, following the procedure of previous CHR investigations (Shim et al., 2008; Phillips et al., 2009; Fusar-Poli et al., 2010; Wood et al., 2011; Rietdijk et al., 2012; Thompson et al., 2012; van der Gaag et al., 2012; McGorry and van Os, 2013; Valmaggia et al., 2013).

The CHR patients were not informed about their at-risk for psychosis status, to not unnecessarily alarm them, since most of them will not make the transition to psychosis. They were told they had ‘extraordinary or unusual experiences’, when discussing psychotic symptoms. These were regularly monitored by their treating clinicians. Regardless of transition rates, the presence of psychotic symptoms in these patients is associated with a poorer prognosis, showing that these patients are in need of special care (Ruhrmann et al., 2010; van Os and Linscott, 2012; McGorry and van Os, 2013; Valmaggia et al., 2013; van Os and Reininghaus, 2016). Further, FEP symptom severity was rather mild, possibly due to responsiveness to antipsychotic treatment. Similar symptom severity has been found in stable and medicated patients (Möller et al., 2005), but a wider range of symptoms might have revealed more associations with social mindfulness at the behavioral or neural level. Additionally, participants were scanned for about an hour, which could have caused fatigue, which may have affected neural outcomes, especially in patients with a psychotic disorder. Questions remain about the motivation for choosing socially (un)mindfully. For further research we recommend additional measures, such as a questionnaire after the task, to inquire after the motivation of participants’ choices; measures of hostility toward other people; and tasks that could rule out the alternative explanation that FEP might encounter choosing the single option as the prepotent, automatic response [see also (Yamagishi et al., 2016)]. Despite controlling for WAIS vocabulary, questions about the association between social mindfulness and verbal and cognitive ability remain. This warrants further investigation.

## Conclusion

This study is the first to examine social mindfulness in patients with problems in social cognition and functioning. Our results show that relative to the healthy control group, spontaneous social mindfulness seems reduced when patients have experienced a first full-blown psychosis. At the same time, social mindfulness was not lower for those at risk for psychosis (CHR). However, when explicitly told to act in the other person’s best interest, FEP are just as capable to be socially mindful as anyone else. Neural outcomes suggest reduced feelings of reward during socially mindful decisions in FEP, and possibly a stronger, automatic inclination to focus on the unique options that seem most attractive for themselves in FEP and CHR. Left to themselves, FEP seem to have reduced appreciation for the more subtle social consequences of leaving or limiting choices. In all, the current research can be seen as a first step in showing reduced socially mindful behavior in psychosis. This aspect of social interactions may possibly underlie deficits in more complex cooperative interactions, such as trust, that patients might otherwise develop within their social environment. Alternatively, displays of low socially mindful behavior may elicit reduced feelings of trust in the counterpart, who in turn will behave less trusting. The next step is to investigate whether and how social unmindfulness serves as a cause underlying patients’ low levels of trust.

## Author Contributions

IL-J collected and processed the data, and wrote the manuscript. LK and PVL designed the study. PVL and NVD designed the paradigm. NVD prepared it for fMRI. DV planned the fMRI analyses. IL-J, A-KF, LK, and DV interpreted the fMRI results. LK supervised the project. All authors discussed the results, contributed to the writing process, and approved the final manuscript.

## Conflict of Interest Statement

The authors declare that the research was conducted in the absence of any commercial or financial relationships that could be construed as a potential conflict of interest.

## Footnotes

1 http://www.quantitativeskills.com/sisa/calculations/bonfer.htm
