# Supplementary material for: Social Mindfulness and Psychosis: Neural Response to Socially Mindful Behavior in First-Episode Psychosis and Patients at Clinical High-Risk
Source: Front Hum Neurosci. 2019 Feb 13;13:47. doi: 10.3389/fnhum.2019.00047 (PMC6381043; doi:10.3389/fnhum.2019.00047)
Supplement: Supplementary file 1 [file Table_1.DOCX]

Supplementary material

Social Mindfulness and Psychosis:

Neural response to socially mindful behavior in first-episode psychosis and patients at clinical high-risk

Imke L. J. Lemmers-Jansen, Anne-Kathrin J. Fett,

Niels J. Van Doesum, Paul A. M. Van Lange, Dick J. Veltman, and Lydia Krabbendam

Supplementary Table S1

Global Null analyses for the conditions of the Social Mindfulness paradigm

| Condition | Region Hemisphere | | Cluster size | | MNI Coordinates | | | | | | | | | | | Z | |  |  |  |  |
| --- | --- | --- | --- | --- | --- | --- | --- | --- | --- | --- | --- | --- | --- | --- | --- | --- | --- | --- | --- | --- | --- |
|  |  | |  | | | | x | | y | | | z |  | |  |  |  |  |  |  |  |
| *Spontaneous Mindful Choices* | | | |  | | | | | | |  | | | | | |  | | |  |  |
|  | dlPFC | R | | 27 | | 36 | | 23 | | 46 | | | | 5.56 | | | | |  |  |  |
|  |  | | |  | | 36 | | 17 | | 37 | | | | 5.38 | | | | |  |  |  |
|  |  | | |  | | 42 | | 14 | | 46 | | | | 5.21 | | | | |  |  |  |
|  | Frontal superior gyrus | R | | 21 | | 18 | | 23 | | 55 | | | | 5.50 | | | | |  |  |  |
|  | TPJ | R | | 324 | | 54 | | -40 | | 52 | | | | 7.50 | | | | |  |  |  |
|  |  | | |  | | 57 | | -46 | | 40 | | | | 6.91 | | | | |  |  |  |
|  |  | | |  | | 42 | | -52 | | 40 | | | | 6.61 | | | | |  |  |  |
|  | TPJ | L | | 18 | | -42 | | -52 | | 43 | | | | 5.12 | | | | |  |  |  |
|  |  |  | |  | | -48 | | -43 | | 40 | | | | 4.83 | | | | |  |  |  |
|  | Precuneus | R | | 31 | | 6 | | -70 | | 40 | | | | 5.76 | | | | |  |  |  |
| *Spontaneous Unmindful Choices* | | | |  | |  | |  | |  | | | |  | | | | |  |  |  |
|  | mPFC | L | | 10 | | -6 | | 41 | | 46 | | | | 5.95 | | | | |  |  |  |
|  | mPFC | R | | 1 | | 9 | | 50 | | 31 | | | | 4.77 | | | | |  |  |  |
|  | vlPFC | L | | 2 | | -45 | | 38 | | -14 | | | | 5.41 | | | | |  |  |  |
|  | vlPFC | L | | 1 | | -30 | | 14 | | -23 | | | | 4.79 | | | | |  |  |  |
|  | vlPFC | R | | 2 | | 45 | | 35 | | -11 | | | | 4.95 | | | | |  |  |  |
|  | vlPFC | R | | 1 | | 57 | | 20 | | 16 | | | | 4.80 | | | | |  |  |  |
|  | Insula | R | | 20 | | 39 | | 17 | | -8 | | | | 5.76 | | | | |  |  |  |
|  |  | | |  | | 42 | | 26 | | -8 | | | | 5.46 | | | | |  |  |  |
|  | Insula | R | | 1 | | 30 | | 20 | | -14 | | | | 5.47 | | | | |  |  |  |
|  | Insula | L | | 4 | | -27 | | 20 | | -14 | | | | 5.41 | | | | |  |  |  |
|  | Superior temporal pole | L | | 2 | | -42 | | 17 | | -14 | | | | 5.31 | | | | |  |  |  |
|  | Inferior parietal gyrus | L | | 3 | | -54 | | -55 | | 46 | | | | 5.26 | | | | |  |  |  |
|  | TPJ | L | | 3 | | -51 | | -58 | | 34 | | | | 5.02 | | | | |  |  |  |
|  | TPJ | L | | 1 | | -57 | | -61 | | 34 | | | | 4.84 | | | | |  |  |  |
|  | Caudate | R | | 7 | | 12 | | 8 | | 7 | | | | 5.01 | | | | |  |  |  |
|  | Caudate | L | | 4 | | -12 | | 2 | | 13 | | | | 5.42 | | | | |  |  |  |
|  | Mid cingulum | L | | 3 | | -3 | | -13 | | 34 | | | | 5.41 | | | | |  |  |  |
| *Mindful Choices after Instruction* | | | |  | |  | |  | |  | | | |  | | | | |  |  |  |
|  | mPFC | R | | 2 | | 9 | | 44 | | 49 | | | | 5.04 | | | | |  |  |  |
|  | mPFC | R | | 3 | | 12 | | 32 | | 55 | | | | 4.93 | | | | |  |  |  |
|  | vlPFC | R | | 1 | | 30 | | 17 | | -20 | | | | 4.96 | | | | |  |  |  |
|  | TPJ | L | | 14 | | -54 | | -55 | | 43 | | | | 6.11 | | | | |  |  |  |
|  | TPJ | R | | 21 | | 60 | | -49 | | 37 | | | | 5.34 | | | | |  |  |  |
|  |  | | |  | | 57 | | -55 | | 43 | | | | 5.11 | | | | |  |  |  |
|  | TPJ | R | | 1 | | 54 | | -64 | | 34 | | | | 4.85 | | | | |  |  |  |
|  | TPJ | R | | 2 | | 57 | | -61 | | 25 | | | | 4.83 | | | | |  |  |  |
|  | TPJ | R | | 1 | | 51 | | -61 | | 31 | | | | 4.80 | | | | |  |  |  |
|  | Precuneus | R | | 2 | | 3 | | -67 | | 43 | | | | 4.99 | | | | |  |  |  |

*Note*: MNI = Montreal Neurological Institute; dlPFC = dorsolateral prefrontal cortex; TPJ = temporo-parietal junction; mPFC = medial prefrontal cortex; vlPFC = ventrolateral prefrontal cortex; L = left; R = right. All analyses were whole brain FWE corrected, showing regions activated by the three participant groups: first-episode psychosis patients, patients at clinical high-risk for psychosis, and healthy controls.
